# Impressive Impact of Hemp Extract on Antioxidant System in Honey Bee (*Apis mellifera*) Organism

**DOI:** 10.3390/antiox11040707

**Published:** 2022-04-02

**Authors:** Patrycja Skowronek, Łukasz Wójcik, Aneta Strachecka

**Affiliations:** Department of Invertebrate Ecophysiology and Experimental Biology, University of Life Sciences in Lublin, Doświadczalna 50a, 20-280 Lublin, Poland; lukasz7wojcik@gmail.com (Ł.W.); aneta.strachecka@up.lublin.pl (A.S.)

**Keywords:** bees’ resistance, cannabis, supplementation of bees, antioxidants

## Abstract

We examined the effect of hemp extract on the activity of the antioxidant system (catalase, peroxidase, glutathione, superoxide dismutase, and total antioxidant capacity) in the hemolymph of adult honey bees (*Apis mellifera*). The bees were divided into three groups: (1) an experimental group fed with pure sugar syrup with cotton strips soaked with hemp extract put inside the cage; (2) an experimental group fed with a mixture of sugar syrup with hemp extract; and (3) a control group fed with a mixture of sugar and a water–glycerine solution. Hemolymph samples were collected on the 1st day of this study and then every week, until all bees in the group died. The activities of all antioxidant enzymes were higher for the experimental groups, compared to those for the control group. The highest antioxidant activities were noted in the group supplemented with cannabis with the use of syringes. Supplementation with hemp also increased the lifespan of bees in this group compared to that of the bees consuming only sugar syrup (control: 35 days), with 49 and 52 days for groups of cannabis on strips and in syrup, respectively. Hemp extract, thanks to its antioxidant properties, increased the activities of key antioxidant enzymes that protect the bee’s organisms against free radicals and thus delay the aging processes.

## 1. Introduction

In recent years, it has been observed the intensification of changes in the environment caused by progressive pollution, which has a negative impact on all living organisms. Despite many activities aimed at protecting the environment, the effects of these changes are observed in many animals, including beneficial insects, such as honey bees [1,2]. Nowadays, high declines in populations of this pollinator and increased susceptibility to infection by specific parasites and pathogens are being recorded [3]. Chemical compounds, i.e., pesticides, are main pollutants that also affect the health of bees [4]. In addition, a general phenomenon of weakening the immune system of bees may be the reason for higher mortality among pollinators [5,6].

Factors negatively affecting the organism (including its immunity) cause strong reactions, often through the increased production of free radicals responsible for chronic inflammation and premature aging of the body [7,8]. The antioxidant system is responsible for protecting against these molecules and, thanks to the activity of its enzymes, neutralizes reactive oxygen species (ROS) produced by organisms. However, due to the increase in the amount of negative factors, a delay in the response of defense systems or too much excitability is observed in a short time, which can lead to autoimmune diseases [9,10,11]. 

In order to additionally strengthen the organism, medicine commonly tests new sources of antioxidants to support animal organisms. Such sources include spices (e.g., curcumin), vegetables (e.g., pumpkin rich in beta-carotene), fruits such as currants (vitamin C), grapes (resveratrol), or dietary additives such as spirulina [12,13,14,15]. These substances/products affect not only the human body, but have also been used as supplementation supporting the health of honey bees [12,13,14,15,16,17]. The addition of curcumin to sugar syrup results in the change of key parameters of immunity in bees fed with this formula, including an increase in the activities of antioxidant enzymes (superoxide dismutase (SOD), peroxidase (GPx), catalase (CAT), glutathione S-transferase (GST), and total antioxidant capacity (TAC)) [13]. The administration of resveratrol extends the life of bees by approximately 33–38%, despite keeping bees under hyperoxic stress conditions [14]. Another product tested on bees is spirulina, which influences physiological parameters (weight) and increases the contents of proteins and lipids in individual parts of the body [12]. 

In this publication, we checked hemp extract which, according to the reports from the fields of human and animal medicine, has a proven antioxidant effect and a number of other properties thanks to which it is widely used in the treatment of many diseases such as epilepsy, Crohn’s disease, depression, and Alzheimer’s disease [18,19,20,21]. Hemp is also used as a supplement in treating cancer or in counteracting the effects of chronic stress/trauma [22]. Its positive effect is attributed to the active substances from the group of cannabinoids (cannabidiol (CBD), cannabichromene, cannabigerol, Δ9-tetrahydrocannabinol (THC), and cannabinol) and the content of phytochemicals [21,23]. During in vitro studies with human cell lines, the aqueous cannabis extract showed a protective effect against the cytotoxicity and apoptosis of the tested fibroblasts and keratinocytes [24]. The other researches involved also other animals. The usage of isolated rat skin confirmed the anti-inflammatory effect of cannabis by reducing the production of prostaglandin E2 and positively changed the values of tryptophan and kynurenine [24]. The latest research also confirms the effect of the extracts on neurological ailments, i.e., chronic pain, thanks to enriching the diet of rodents with hemp oil and palmitoylethanolamide, which together creates antinociceptive effects [18]. The effect on the increase of interleukin transcription (the amount of mRNA), which takes part in numerous processes of the immune system and mediates information in the body, was also noticed [18]. Moreover, cannabis essential oils prolong the life of *Galleria mallonela* by limiting the invasion of *Listeria monocytogenes*. This confirms that the extract, at appropriate concentrations, is not dangerous to insects [25]. Like humans, bees are also exposed to ROS in their bodies. Therefore, the enzymes present in humans can also be tested in bees. There are four main enzymes that make up the basic first lines of defense against oxidants: SOD, CAT, GPx, GST, and TAC [9,26,27,28,29]. The task of antioxidant enzymes is to trap oxygen radicals and transform them into less reactive forms. SOD participates in the first reactions with radicals, converting them into hydrogen peroxide and molecular oxygen. Then, the hydrogen peroxide is reduced to molecular oxygen and water by CAT or by GPx while reducing glutathione to its oxidized form. GST participates in the changes that detoxify oxygen (the second line of defense). TAC tells us about the overall capacity of the antioxidants present in each organism. Due to the confirmed antioxidant properties of cannabis, we assumed that the extract would have a positive effect on the activity of the enzymes of the antioxidant system: SOD, CAT, GPx, GST, and TAC [9]. 

The aim of our research was to determine the effect of hemp extract on the activities of enzymes in the antioxidant system in bees’ hemolymph, along with the aging processes.

## 2. Materials and Methods

### 2.1. Sampling

The experiment began with 1-day-old worker bees (*A. mellifera carnica*), collected from the apiary belonging to the University of Life Sciences in Lublin (51°13′31″ N, 22°38′07″ E). The colonies were not treated against *Varroa destructor* (*V. destructor*). The treatment was not applied, because the substances used against *V. destructor* could be an additional factor influencing the level of the parameters activities of the immune system. The introduction of this factor would not allow a clear assessment of the effect of the hemp extract. We did not record any spores of *Nosema* spp. in the bees of the colony. The bees in the colonies were in good health. Five mother queens were raised from larvae hatches of eggs laid by one purebred reproductive queen *A. mellifera carnica*. This queen was earlier artificially inseminated. The queen was restricted on one comb in a queen-excluder comb-cage containing one empty comb for 12 h, where laid eggs. After that, the queens were released to the colony, and 4 days after the release, we grafted the larvae from the comb in artificial queen cells. The grafted larvae were placed on a drop of a royal jelly solution (*v*:*v*, 1:1) in the center of the queen cell. The frames with the grafted cells were placed in a 10-frame-strong, queenless colony. After 7 days, the queen cells were isolated and kept in an incubator (35 °C) until emergence. Five of these mother queens were subjected, in Eppendorf tubes (1.5 mL) with cut ends and sealed with wax, to previously prepared five queenless colonies kept in Dadant hives. Queens on the 8th day of their life were artificially inseminated with the semen of *A. mellifera carnica* drones. These males, at 18–20 days of age, were collected from other colonies of the experimental apiary. They were not brothers of the queens. After about a month, the queens were confined to a queen-excluder comb-cage containing one empty comb for 12 h to lay eggs. After 20 days, from which time the queens laid their eggs, the combs were transferred to an incubator, where the workers (1-day-old workers) were emerged. These workers were randomly placed into 30 wooden cages (40 bees per cage) and divided into three groups (10 cages each). The cages were divided into three groups according to Table 1 [4,30].

The cages were kept in optimal conditions of 35 °C and a 65% relative humidity [30]. 

Water–glycerine solutions were brought/delivered from Chempur (standard: BN-76/6193-12; Piekary Śląskie, Poland). The hemp extract used in this test/research was sourced from the manufacturer Melisa (Brzeziny, Poland). 

In each group, food was administered ad libitum and replenished every other day during the experiment.

Ten randomly selected cages were allocated to each group. The analytical procedure, described below, began two days after the first feeding.

### 2.2. Hemolymph Extraction

In each group, fresh hemolymph was taken from 10 bees at the age of 1, 7, 14, 21, 28, 35, 42, 49, and 56 days (by puncturing the venous sinus in the insect’s abdomen), according to Łoś and Strachecka method [10]. The hemolymph from each bee was placed separately in a sterile Eppendorf tube with 0.6% NaCl. All samples (from the control group: 6 sampling × 10 workers; from the experimental group with a strip: 8 sampling × 10 workers; from the experimental group with a syringe: 9 sampling × 10 workers) were immediately refrigerated at −25 °C for further biochemical analyses. The number of sampling depended on the presence of living bees in a given group [31]. 

### 2.3. Determination of Antioxiant Activities

− SOD according to Podczasy and Wei (1988)

The analyzed biological material was hemolymph. We added 0.5 mM xanthine, 0.3 mM EDTA, 49 mM p-iodonitrotetrazolium, and 0.92 mM Na_2_CO_3_ to an Eppendorf tubes. Then, 5 µL of hemolymph were added to the mixture. Reactions were started by adding a maximum of 0.25 U/mL xanthine oxidase. Eppendorf tubes were subjected to a 15-min incubation at 25 °C and then cooled. In the next step, the absorbance was measured at 505 nm.

− GPx according to the methods described by Chance and Maehly (1955)

The analyzed biological material was hemolymph. The Eppendorf tube contained 25 μL assay mixture of 125 μM phosphate buffer (pH 6.8), 50 μM pyrogallol, 50 μM H_2_O_2_, and 5 μL of the biological material sample. The samples were incubated for 5 min at 25 °C. In the next step, the reaction was stopped by adding 5 µL of 5% H_2_SO_4_ to the mixture. Absorbance was measured at a wavelength of 420 nm.

− CAT according to Aebi (1983)

The biological material used in the analyses was hemolymph. Three-hundred and thirty-five microliters of 50 mM phosphate buffer (pH 7.0) were mixed with 165 µL of 54 mM H_2_O_2_ at 25 °C. The reaction was initiated by adding 5 µL of the biological material. One unit of CAT is the amount of the enzyme that decomposes 1 μmol of H_2_O_2_ per minute at 25 °C. The H_2_O_2_ decomposition was measured at a wavelength of 240 nm.

− GST according to Warholm et al. (1985)

The biological material used in the analysis was hemolymph. The following mixture was added to a cuvette: 215 µL 0.1 M sodium phosphate buffer (pH 6.5), 13 µL 20 mM GSH, and 13 µL 20 mM 1-chloro-2,4-dinitrobenzene in 95% ethanol. The reaction was initiated by supplementing the mixture with 12 µL of the biological material. The reaction was carried out at a temperature of 30 °C. Absorbances were measured at 340 nm.

− TAC according to the protocol included in the Assay Kit produced by Sigma Aldrich. The reaction was carried out at a temperature of 30 °C. Absorbances were measured at 570 nm.

All antioxidant enzyme activities were calculated per 1 mg of protein. 

### 2.4. Statistical Analysis

The results were analyzed using Statistica formulas version 13.3 (2017) for Windows (StatSoft Inc., Tusla, OK, USA). The mixed-model two-way ANOVA followed by Tukey HSD post hoc tests (*p* = 0.05) was used to compare the results for each antioxidant enzymes (SOD, GST, CAT, GPx, and TAC) of honey bee workers, depending on the method of administration (strip and syringe) and the day (1st, 7th, 14th, 21st, 28th, 35th, 42nd, 49th, and 56th) of the supplementation with hemp extract.

## 3. Results

**Figure 1:**
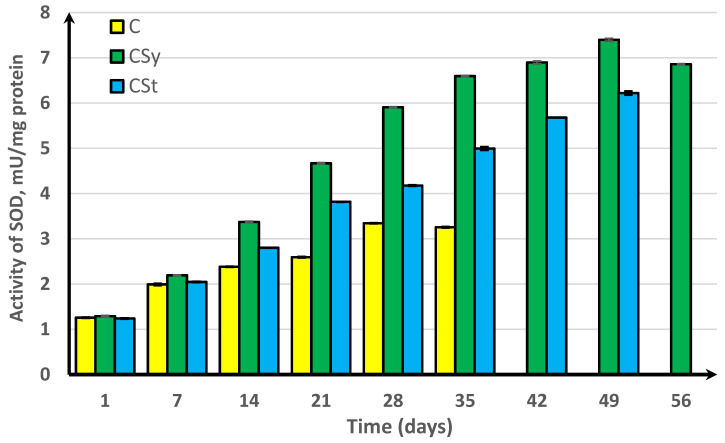
Superoxide dismutase (SOD) activities in workers’ hemolymph along with the aging processes, after using two methods of supplementation with hemp extract. C, control (pure sugar syrup); CSy, hemp extract in syrup; CSt, hemp extract on strips (two-way ANOVA; days of supplementation: F_(5,1078)_ = 17,089, *p* = 0.0000, se ± 1.26111 (1st day), se ± 2.07672 (7th day), se ± 2.85162 (14th day), se ± 3.69264 (21st day), se ± 4.47489 (28th day), se ± 4.94804 (35th day), se ± 6.89712 (42nd day), se ± 6.80810 (59th day), and se ± 6.85724 (56th day); supplementation method: se ± 0.00767 (C), se ± 0.00627 (CSy), and se ± 0.00710 (CSt); supplementation method × days of supplementation: F_(11,1078)_ = 1205.8, *p* = 0.000, and se ± 0.01880). The chart shows statistical averages. The standard deviations for the means in this plot ranged from 0.04136 to 0.30656.

**Figure 2:**
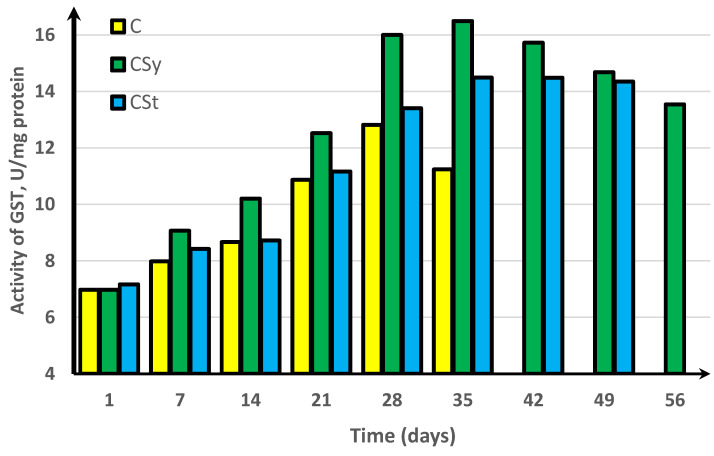
Glutathione S-transferase (GST) activities in workers’ hemolymph along with the aging processes, after using two methods of supplementation with hemp extract. C, control (pure sugar syrup); CSy, hemp extract in syrup; CSt, hemp extract on strips (two-way ANOVA: supplementation method: F_(5,1078)_ = 40,402, *p* = 0.0000, se ± 0.010431 (C), se ± 0.008517 (CSy), and se ± 0.009657 (CSt); days of supplementation: F_(5,1078)_ = 40,402, *p* = 0.0000, se ± 0.014751 (1st–35th days), se ± 0.025550 (42nd day and 56th day), and se ± 0.018066 (49th day); supplementation method × days of supplementation F_(11,1078)_ = 1416.0, *p* = 0.0000, and se ± 0.025550). The chart shows statistical averages. The standard deviations for the means in this plot ranged from 0.04158 to 0.31201.

**Figure 3:**
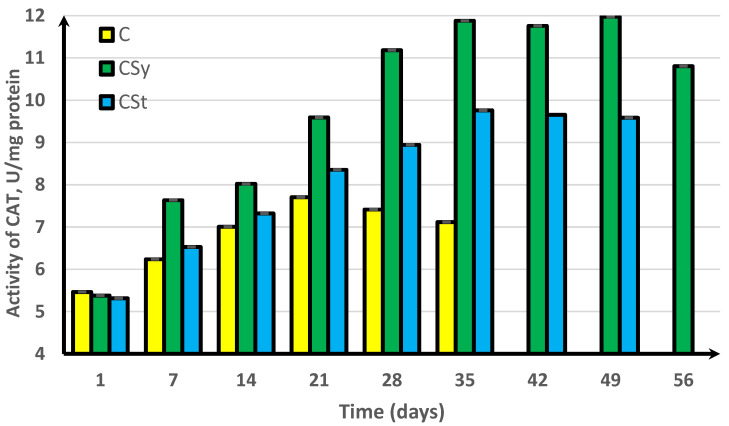
Catalase (CAT) activities in workers’ hemolymph along with the aging processes, after using two methods of supplementation with hemp extract. C, control (pure sugar syrup); CSy, hemp extract in syrup; CSt, hemp extract on strips (two-way ANOVA: days of supplementation: F_(5,1078)_ = 25,133, *p* = 0.0000, se ± 0.010013 (1st–35th days), se ± 0.017343 (42nd day and 56th day), se ± 0.012263 (49th day); supplementation method: se ± 0.00708 (C), se ± 0.00578 (CSy), and se ± 0.00656 (CSt); supplementation method × days of supplementation F_(11,1078)_ = 2701.1, *p* = 0.0000, and se ± 0.017343). The chart shows statistical averages. The standard deviations for the means in this plot ranged from 0.05238 to 0.21342.

**Figure 4:**
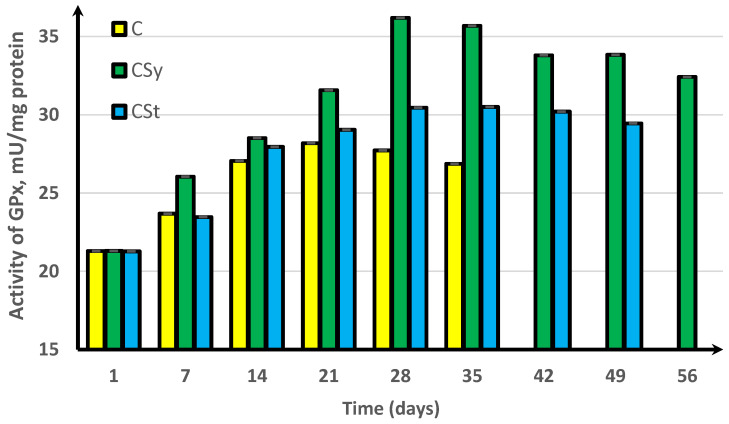
Peroxidase (GPx) activities in workers’ hemolymph along with the aging processes, after using two methods of supplementation with hemp extract. C, control (pure sugar syrup); CSy, hemp extract in syrup; CSt, hemp extract on strips (two-way ANOVA: supplementation method: F_(5,1078_) = 41,905, *p* = 0.0000, se ± 0.013879 (C), se ± 0.011332 (CSy), and se ± 0.012849 (CSt); days of supplementation: F_(5,1078)_ = 41,905, *p* = 0.0000, se ± 0.019628 (1st–35th days), se ± 0.033996 (42nd day and 56th day), se ± 0.024039 (49th day); supplementation method × days of supplementation F_(11,1078)_ = 2910.4, *p* = 0.0000, and se ± 0.033996). The chart shows statistical averages. The standard deviations for the means in this plot ranged from 0.06757 to 0.47376.

**Figure 5:**
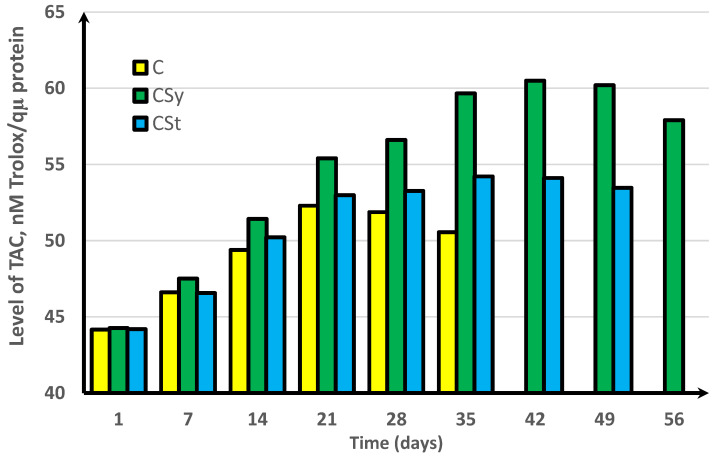
Levels of total antioxidant capacity (TAC) in workers’ hemolymph along with the aging processes, after using two methods of supplementation with hemp extract. C, control (pure sugar syrup); CSy, hemp extract in syrup; CSt, hemp extract on strips (two-way ANOVA: supplementation method: F_(5,1078)_ = 50,034, *p* = 0.0000, se ± 0.011082 (CSy), se ± 0.012565 (CSt), and se ± 0.013572 (C); days of supplementation: F_(5,1078)_ = 50,034, *p* = 0.0000, se ± 0.019194 (1st–35th days), se ± 0.033245 (42nd day and 56th day), and se ± 0.023508 (49th day); supplementation method × days of supplementation: F_(11,1078)_ = 2952.4, *p* = 0.0000, and se ± 0.033245). The chart shows statistical averages. The standard deviations for the means in this plot ranged from 0.10133 to 0.35040.

In the experimental groups, we noticed a continuous increase in activity up to the 42nd day of the life of bees. For comparison, the bees that did not receive supplementation only survived for 35 days (Figure 1, Figure 2, Figure 3, Figure 4 and Figure 5).

In the group where the extract was administered in a syringe, we observed the highest values of the enzyme activity, compared to in the other groups. The bees that had the extract strip in the cages survived longer (49 days) than those in the control group (35 days) but shorter than the bees in the syrup extract group (56 days) (Figure 1, Figure 2, Figure 3, Figure 4 and Figure 5).

In the control group, the activities of antioxidant enzymes increased by day 28 for SOD (Figure 1) and GST (Figure 2) and by day 21 for CAT (Figure 3) and GPx (Figure 4). Then, these values decreased. In the groups supplemented with hemp extract, all tested enzymes achieved higher activities compared to those in the control groups.

## 4. Discussion

Hemp extract visibly increased the activity of antioxidant enzymes in the bees that were fed with this additive, compared to in the bees in the control group. 

The enzymes indicated in our work, i.e., SOD, GST, CAT, GPx, and TAC, are key to the defense of the organism of invertebrates against ROS, and as a consequence of the action of free radicals, they combat oxidative stress. Significant differences between both experimental groups and the control group in activities were demonstrated in the SOD, CAT, and GPx enzymes, which constitute the primary internal antioxidants that complete the defense system. SOD is responsible for the first transformation of free radicals into hydrogen peroxide and oxygen molecules. The hydrogen peroxide formed in the reaction is then converted by CAT into molecular oxygen and water. The enzyme GPx, which has the ability to reduce both organic and inorganic peroxides, also takes part in the reduction of hydrogen peroxide. GST is involved in detoxification by catalyzing the fusion reaction of glutathione with other endogenous and exogenous compounds (e.g., xenobiotics) [13]. TAC gives information about the total nonenzymatic antioxidant capacity of the capability to counteract ROS.

The increase in the activities of antioxidant enzymes could be caused by the influence of CBD on the permeability of ion channels, i.e., potassium, sodium, and calcium, and therefore change in the cell membrane environment. Changes in ion concentration may induce an increase in antioxidants and thus also induce an increase in catalase, which is synthesized in the liver of mammals, depending on the amount of H_2_O_2_, the level of which relies on the amount of calcium in the cytosol [32,33]. High concentrations of antioxidants in insects are usually observed in metabolically active tissues, i.e., the fat body that functions as the liver. This may indicate that the synthesis of antioxidants may be dependent on the processes that take place in vertebrates in the organs equivalent to those of invertebrates [9].

On the other hand, many studies suggest that an increased mRNA expression of genes corresponding to the antioxidant activity in insects depends on external factors (including stress factors) that cause ROS formation. We suggest that the activities of antioxidants are modified and depend on the number of oxidizing agents with which the organisms of insects meet. This may suggest that by administering a foreign substance (cannabis) to the bees, we caused a temporary increase in oxidative stress. This enabled us to stimulate higher mRNA expression in the early stages of life, which in the later age of the bees allowed for a better/more intense reaction to standard ROS (higher enzyme activities in the early stage of life) [34,35]. 

The antioxidant activity of cannabis has so far been described in studies that used seeds and oils. The studies on the hydrolyzate of proteins from hemp seeds (obtained during in vitro digestion) showed significant antioxidant properties (up to 67% of the radical scavenging activity, i.e., 2,2-diphenyl-1-picrylhydrazyl), metal chelating (activity up to 94%), and Fe^3+^ reduction. It owes its properties to the peptide fractions: Trp-Val-Tyr-Tyr (tetrapeptide) and Pro-Ser-Leu-Pro-Ala (pentapeptide). Additionally, fractionated peptides showed higher chelating activity than glutathione in studies [36,37]. Such a strong radical scavenging process may be related to the extension of the life of bees in our experiment (given the great antioxidant effects for various forms of the processed hemp raw materials). Similar properties have been demonstrated for the essential oil in relation to 2,2-diphenyl-1-picrylhydrazyl, and β-carotene/linoleic acid tests and Fe^3+^-reducing properties have been reported [38]. Hemp owes much of its properties to the active substances from the cannabinoid group. The antioxidant potential of the oils has been confirmed and measured in tests in which the main active substances were CBD, THC, or compounds from the group of flavonoids and terpenes [39]. Due to the use of an oil consisting mainly of CBD in our research, we looked for experimental results of this compound in the literature. Hacke et al. [16] showed a remarkable antioxidant activity for CBD during spectrophotometric and electrochemical analysis, which we also showed in our experiment with the bees consuming hemp extract [16]. During the research, the activity of this active compound was compared with the properties of other antioxidant compounds, i.e., resveratrol. Resveratrol has also been tested in bee supplementation and has also shown a positive effect on antioxidant activity. Interestingly, according to the research, CBD has a stronger scavenging capacity for 2,2-diphenyl-1-picrylhydrazyl radicals than pure THC. CBD owes its strong action to its chemical structure: it has two phenolic groups responsible for the antiradical function [16]. Phenolic antioxidants (group of polyphenols) create nonradical products as a result of their transformation from superoxide radicals [40]. Such strong activities of antioxidants in the hemolymph of the bees fed with the supplement can multiply/intensify the beneficial effects of phenolic groups and thus extend the life of these pollinators. 

The antioxidant activities of compounds belonging to polyphenols were also demonstrated by other compounds used in bee supplementation, i.e., the previously mentioned curcumin, coenzyme Q10, caffeine, resveratrol, and piperine [13,14,41,42]. All publications have reported a positive effect of plant metabolites on increasing the activities of the antioxidant system enzymes and/or significantly extending the life of bees in the groups fed with the supplement. The trends observed in the cannabis extract tests are similar to the rest of the tests conducted by Strachecka on curcumin, coenzyme Q10, caffeine, and piperine [11,13,41,42]. The addition of hemp extract stood out from other studies as it extended life up to day 56, while other publications report 48 days for turmeric, 41 days for piperine, and 38 days for coenzyme Q10. Additionally, in our study, we tested two key supplementation methods used in laboratories (syrup in a syringe) and in hives on an apiary (strips). The purpose of using two methods was to determine whether the method of administration had an effect on the performance of the biostimulator. As it was found, the syringe method of supplementation had better results compared to the strip method. We suspect that this is related to the longer time it takes for the extract from the strip to enter the bees’ organism. The positive effect was visible in insects consuming cannabis directly through the syrup, which allowed for the maximum use of its potential and direct distribution in the body. The extract placed on the strips probably made its way into the bees’ digestive system and then to other tissues by steaming and sticking to the particles of the bodies of bees that then were licked by other workers during trophallaxis. 

## 5. Conclusions

Hemp extract significantly increased the activity of antioxidant enzymes, extending the life of bees to 49 days (for the strip method) and 56 days (for the syringe method). In addition, we showed that a faster and stronger effect was obtained during supplementation in syrup in syringes, where the activities for the enzymes SOD, CAT, GPx, GST, and TAC were the highest. Thanks to this, we believe that hemp extract can in the future contribute to the improvement of the natural immunity of honey bees and help them with the fight against environmental pollution and the increase of oxidative stress.

## Figures and Tables

**Table 1:** Descriptions of supplementation and administration methods for the group in the experiment.

Type of Group	Feeding	Method of Supplementation
(1) CSt	sugar syrup (*v*:*v*, 1:1) ad libitum and inside with cotton strips soaked with 3 mL hemp extract (0.25 g hemp paste extract + 3 mL water–glycerine solution)	extract on a cotton strip
(2) CSy	a mixture of sugar syrup (*v*:*v*, 1:1) with hemp extract ad libitum (500 mL water–glycerine solution with 4.38 g hemp paste extract)	extract in a syringe
(3) C	mixture of sugar and a water–glycerine solution (*v*:*v*, 1:1)	syrup in a syringe

## Data Availability

The datasets and materials that have been used, analyzed and presented in this manuscript are not publicly available but available at the University of Life Sciences in Lublin. At a justified request of the interested party, they may be made available by the corresponding author.
